# Human immunodeficiency virus type 1 envelope proteins traffic toward virion assembly sites via a TBC1D20/Rab1-regulated pathway

**DOI:** 10.1186/1742-4690-9-7

**Published:** 2012-01-19

**Authors:** Dikla Nachmias, Ella H Sklan, Marcelo Ehrlich, Eran Bacharach

**Affiliations:** 1 Department of Cell Research and Immunology, The George S. Wise Faculty of Life Sciences, Tel Aviv University, Tel Aviv 69978, Israel; 2 Department of Clinical Immunology and Microbiology, Sackler School of Medicine, Tel Aviv University, Tel Aviv 69978, Israel

**Keywords:** HIV-1, envelope, assembly, TBCID20, Rab1, secretory pathway

## Abstract

**Background:**

The cellular activity of many factors and pathways is required to execute the complex replication cycle of the human immunodeficiency virus type 1 (HIV-1). To reveal these cellular components, several extensive RNAi screens have been performed, listing numerous 'HIV-dependency factors'. However, only a small overlap between these lists exists, calling for further evaluation of the relevance of specific factors to HIV-1 replication and for the identification of additional cellular candidates. TBC1D20, the GTPase-activating protein (GAP) of Rab1, regulates endoplasmic reticulum (ER) to Golgi trafficking, was not identified in any of these screens, and its involvement in HIV-1 replication cycle is tested here.

**Findings:**

Excessive TBC1D20 activity perturbs the early trafficking of HIV-1 envelope protein through the secretory pathway. Overexpression of TBC1D20 hampered envelope processing and reduced its association with detergent-resistant membranes, entailing a reduction in infectivity of HIV-1 virion like particles (VLPs).

**Conclusions:**

These findings add TBC1D20 to the network of host factors regulating HIV replication cycle.

## Findings

Numerous host factors regulate, directly and indirectly, different steps of HIV-1 infection. To reveal these factors, several extensive RNAi screens have been performed, listing over a thousand proteins [1-5]. Surprisingly, there is only a small overlap between these lists, calling for further evaluation of the relevance of specific factors to HIV-1 replication [6,7]. The small GTPase Rab1 has been marked in one of these screens as a putative HIV-dependency factor [1]. Here we identified TBC1D20, a specific GAP of this Rab [8,9], as a new host factor that regulates HIV replication.

Rab1, which cycles between active GTP-bound and inactive GDP-bound forms [10], and is present as Rab1a/b isoforms, regulates the early secretory pathway by controlling ER to Golgi traffic [11]; yet, unconventional Rab1-independent secretion pathway(s) have also been described [12-14]. TBC1D20, a Rab1-GAP, inactivates Rab1 through the stimulation of GTP hydrolysis; accordingly, TBC1D20 overproduction blocks Rab1-mediated ER-to-Golgi transport [8,9]. The utilization of the Rab1-dependent secretory pathway by HIV-1 and the influence of TBC1D20/Rab1 axis on its infectivity remain unexplored.

**Figure 1:**
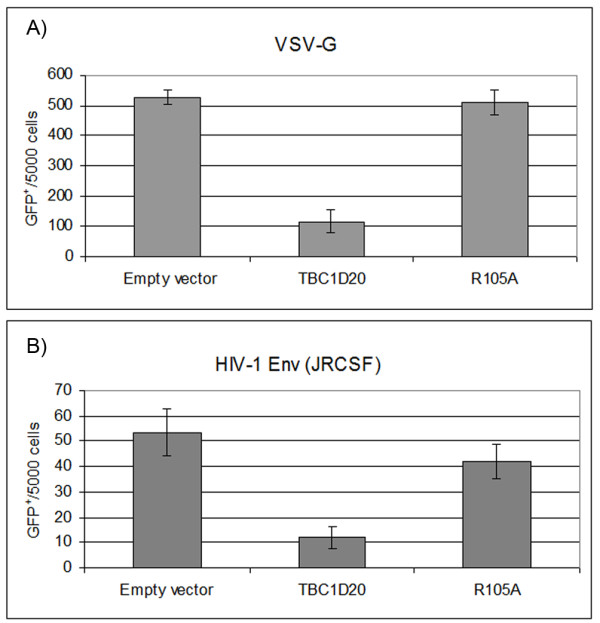
**Overexpression of TBC1D20 decreases HIV-1 VLPs infectivity**. HIV-1 VLPs, pseudotyped with either VSV-G (A) or JRCSF (B) glycoproteins, were generated in 293T cells (~5 × 106 cells/60 mm plate), overexpressing TBC1D20, R105A, or empty vector. HeLa TZM-bl cells were infected with equal amounts of these VLPs and the number of GFP^+ ^cells per 5,000 cells was determined by FACS. The data are represented as the mean ± the standard error of the mean (SEM, *n *= 3).

The maturation and assembly of HIV-1 envelope (Env) occur along the secretory pathway. Env maturation requires post-translational modifications, including the initial acquisition of high-mannose sugar trees that occurs within the ER. Subsequent maturation steps such as the trimming of this sugar-tree, acquisition of different sugars that modify Env apparent molecular weight (MW) and the furin-mediated cleavage of gp160 precursor to gp120 and gp41, require Env transport through posterior secretory pathway compartments [15]. Finally, targeting to specific domains of the plasma membrane is required for efficient assembly of Env into nascent virions [16,17]. Despite extensive study, the full milieu of specific factors and secretory pathways involved in these processes remains unknown. To investigate if Env maturation and trafficking are regulated by TBC1D20 and occur via Rab1-dependent pathway, we exploited the effect induced by imbalanced expression of TBC1D20 on Rab1-mediated transport, and probed for the infectivity of HIV-1 VLPs in such conditions. In previous studies, it was shown that TBC1D20 is a negative regulator of the ER-to-Golgi traffic of a temperature sensitive mutant of the envelope glycoprotein of the vesicular stomatitis virus (VSV-G) in HeLa cells [8,9]. To expand this finding to the present experimental system, 293T cells were co-transfected with plasmids encoding for VSV-G (2.5 μg), the HIV-1 Gag-Pol [ΔR8.2;[18] 7.5 μg], a retroviral vector encoding GFP [pHR'CMV-GFP [19]; 10 μg], and Myc-tagged TBC1D20 [pMyc-TBC1D20 [9]; 10 μg]. A Myc-tagged catalytically inactive form of TBC1D20 [R105A [8,9]; 10 μg)], or an empty vector [pCMV-Myc (Clontech); 10 μg] served as controls. Supernatants were collected 48 h post transfection and equal amounts of VLPs, normalized by RT exogenous assay [20], were used to infect naïve HeLa TZM-bl cells, expressing CD4, CXCR4 and CCR5 molecules [21]. Of note, TZM-bl cells carry the LTR-driven firefly luciferase as well as the β-galactosidase reporter genes to monitor HIV infection; however, we used the GFP reporter in pHR'CMV-GFP vector to quantify infection levels since this vector does not express the Tat protein, which is essential for luciferase and β-galactosidase activation in infected TZM-bl cells; and as GFP expression enables the quantification of large numbers of infected cells through fluorescence-activated cell sorting (FACS) analysis. Figure 1A shows that in accord with a block to VSV-G trafficking, overexpression of TBC1D20 decreased the number of infected cells (GFP+) to 20% of the control. To address if HIV Env is analogously regulated, a similar experiment was performed with HIV Env [JRCSF [22]; 2.5 μg] (Figure 1B). Here too, TBC1D20 overexpression decreased the infectivity of VLPs suggesting that VSV-G and HIV Env traffic through a Rab1-sensitive pathway.

**Figure 2:**
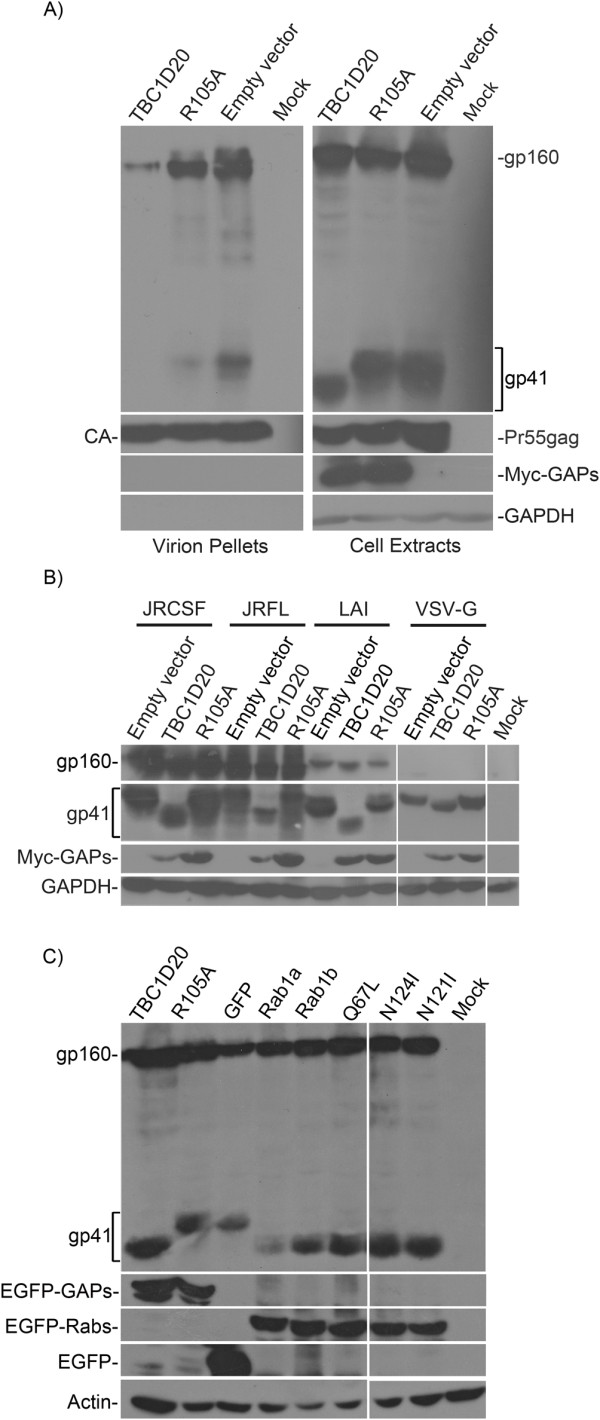
**Overexpression of TBC1D20 alters the apparent MW of Env and decreases its incorporation into VLPs**. (A) HIV-1 VLPs, pseudotyped with JRCSF envelope, were generated in 293T cells (~2 × 106/well, 6-well plate) overexpressing the indicated GAPs (TBC1D20 or R105A) or an empty vector. Cell extracts were prepared from transfected cells and from virion pellets that were purified from the supernatants of the transfected cultures. Western blot was used to detect the expression of Env, Myc-tagged GAPs, CA and GAPDH, using monoclonal antibodies against gp41 [Chessie 8 hybridoma, [37]; Myc epitope (9E10 hybridoma); CA [183-H12-5C hybridoma, [46]] and GAPDH (Chemicon, cat. #MAB374). (B) Identical procedure was used to monitor the effect of overexpression of the indicated GAPs on the apparent MW of gp41 (JRFL, LAI and JRCSF strains) and VSV-G; and (C) to monitor this effect on JRCSF Env upon overexpression of the indicated Rab forms. These forms were expressed as GFP fusions [9], where the parental pEGFP-C1 plasmid (Clonetech), expressing the GFP alone, served as a negative control. Antibodies against GFP (Convance, cat. #MMS-118R) and actin (MP Biomedicals, cat. #69100) were used to detect protein expression.

To further characterize the source of this decrease, 293T cells were transfected with ΔR8.2 (1 μg), JRCSF (1.5 μg) together with pMyc-TBC1D20, R105A or pCMV-Myc (3 μg each). Lysates from the transfected cells and virions purified from the culture supernatants by ultracentrifugation through a 25% sucrose cushion were subjected to Western blot analysis (Figure 2). Similar capsid (CA) levels were observed in all the samples, suggesting that TBC1D20 overexpression did not reduce VLPs production. In contrast, TBC1D20 overexpression greatly decreased the levels of gp160 and gp41 in VLPs (Figure 2A; 'Virion Pellets' panel): the average levels of Env proteins, measured by densitometry in three independent experiments, was 18% ± 7 (gp160) and 0.8% ± 0.5 (gp41), compared to the 100% of the empty vector control. Overexpression of R105A resulted in a smaller reduction (Figure 2A; 'Virion Pellets' panel), with average levels of 80% ± 5 (gp160) and 39% ± 17 (gp41). The reduction in Env virion levels observed for R105A may be explained by the fact that although catalytic inactive, it is still able to interact with other cellular proteins such as the reticulon [8]. This latter protein is localized to the ER, and evidence exist for its influence on ER-to-Golgi trafficking, vesicle formation and membrane morphogenesis [23]. Thus, R105A overexpression may still affect Env levels to some extent in a Rab1-independent manner. In any case, the reduction in the virion Env levels, observed for either TBC1D20 or R105A expression, is in agreement with the decrease in VLPs infectivity (Figure 1B). TBC1D20 overexpression resulted in a faster migrating form of gp41 (Figure 2A; 'Cell Extracts' panel), likely reflecting altered glycosylation (see below). This change in migration was not evident for gp160, as expected for ER-localized protein. While TBC1D20-overexpression altered the migration of gp41 in all experiments (*n *= 15), in some experiments (*n = *7) a small reduction in gp41 level was also observed, probably reflecting a reduction in gp160 cleavage (see for example the LAI Env in Figure 2B, where gp160 level was higher but gp41 level was reduced, in the TBC1D20 sample compared to the controls). Overall, these data suggest that enhanced TBC1D20 activity caused abnormal Env processing and reduced its incorporation into nascent virions, correlating with the reduced infectivity of such particles.

Because of the profound effect of TBC1D20 overexpression on gp41 migration (JRCSF strain), we expanded this analysis to the JRFL (R5 strain) and LAI (X4 strain) envelope proteins [22,24,25] and to the VSV-G. TBC1D20 overexpression resulted in abnormal migration of all these proteins (Figure 2B). This result is in line with TBC1D20 effect on ER-to-Golgi trafficking of VSV-G [8,9] and with the reduced infectivity of HIV-1 VLPs pseudotyped with VSV-G (Figure 1A). Overall, these results indicate the generality of TBC1D20 activity on the cellular processing of the envelope glycoproteins of these viruses. In accord with this conclusion, TBC1D20 overexpression also resulted in abnormal migration of the transforming growth factor beta receptor II (TGFβ receptor II) - a glycosylated cellular protein that traffics through the secretory pathway to the plasma membrane (data not shown).

The above effect of TBC1D20 on the migration of envelope glycoproteins is expected to reflect a lack of Rab1 activity - the target of TBC1D20. We next examined if direct modulations of Rab1 activity will similarly affect gp41 migration. We transfected cells with the JRCSF Env and Gag-Pol proteins (as in Figure 2A) and either TBC1D20, R105A, Rab1a, Rab1b, constitutively-active Rab1b (Q67L) [26] or the dominant negative (DN) forms of Rab1a (N124I) or Rab1b (N121I) [11]. All of these proteins were expressed as GFP fusions [9], accordingly a GFP only negative control was used. All treatments, except for the negative controls (R105A or GFP), induced the aberrant migration of gp41 (Figure 2C). The DN activity of N124I and N121I, similar to TBC1D20 overexpression, reduces Rab1 function and thus the aberrant migration of gp41 is expected. Surprisingly, overexpression of Rab1a, Rab1b or Q67L, which enhances Rab1 activity, also resulted in abnormal gp41 migration. An explanation for this might be the observation that excesses of Rab1 activity increase ER-to-Golgi transport [27], and this too may impair proper glycosylation. Thus, a precise regulation of the Rab1/TBC1D20 axis may be necessary for proper gp41 processing.

**Figure 3:**
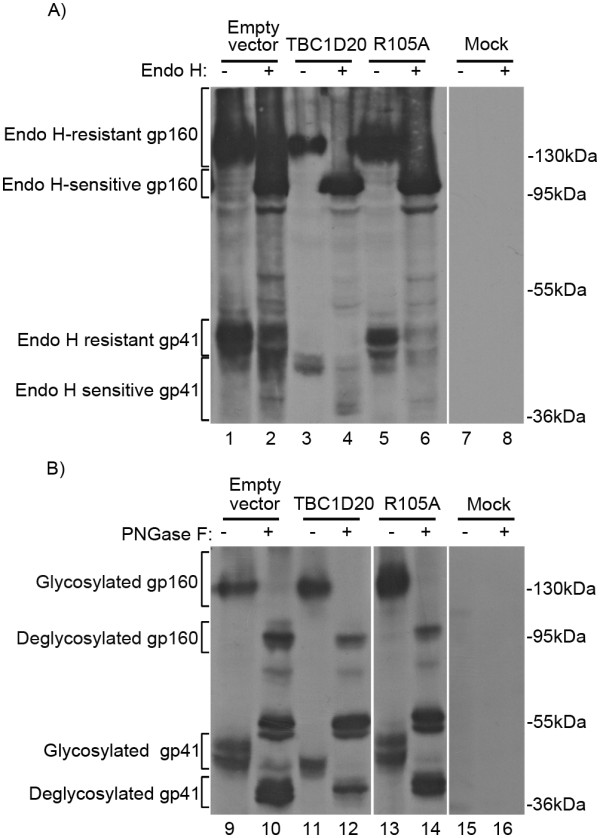
**TBC1D20 overexpression impairs proper HIV Env glycosylation**. Extracts (100 μg of total protein) of 2 × 10^6 ^293T cells expressing HIV-1 Gag-Pol and JRCSF Env proteins, together with either TBC1D20, or controls (R105A or empty vector), were treated with ('+') or without ('-') EndoH (A) or PNGase F (B). Extracts were examined by Western blot analysis using anti-gp41 monoclonal antibody, to detect gp160 and gp41.

Sugar modifications are indicative of protein trafficking through specific compartments of the secretory pathway [28]. Since TBC1D20/Rab1 function in regulating ER-to-Golgi trafficking [8,9,11] and since Env is heavily glycosylated [29], the observed aberrant migration of Env in SDS-PAGE for cells overexpressing TBC1D20 suggests alterations in Env glycosylation and trafficking. To study this, we performed deglycosylation sensitivity assays, as described [30]. 293T cells were transfected as described in Figure 2 and lysed; the extracts were treated with endoglycosidase H (EndoH), which cleaves N-linked high-mannose oligosaccharides, found on proteins located in the ER/cis Golgi [31]. SDS-PAGE-immunoblot analysis of lysates of cells expressing JRCSF Env, using anti-gp41 antibodies, yielded two main forms of Env corresponding to gp160 and gp41 that appeared smeared, suggesting multiple glycosylated forms of Env (see 'empty vector' and 'R105A' controls - Figure 3A, lanes 1 and 5). To confirm that the broad distribution of Env molecules stems from glycosylation, we digested the cell lysates with Peptide:N-glycosidase F (PNGase F), which removes all types of N-linked glycosylation [32]. Indeed, this treatment resulted in the collapse of the smears to distinct bands of approximately 96 and 39 kDa; matching the net MW of these proteins (see controls - Figure 3B, lanes 9, 10, 13 and 14). EndoH treatment resulted in a similar collapse of gp160 forms while failing to markedly alter the distribution of majority of gp41 molecules (see controls - Figure 3A, lanes 1, 2, 5 and 6; 'EndoH-resistant gp41'). These results are in accord with ER localization of gp160 as it is mainly EndoH-sensitive and support the traffic of gp41 up to the trans-Golgi network since it is both furin-cleaved and EndoH-resistant [33]. These results are also in line with previous studies that established that the kinetics of EndoH-resistance closely parallel those of Env cleavage [34]. Next, we addressed the effect of TBC1D20 on Env glycosylation. While all Env forms were equally sensitive to PNGase F (Figure 3B, lanes 11 and 12), and gp160 remained EndoH-sensitive (Figure 3A, lanes 3 and 4), a clear reduction in the apparent MW of gp41 was observed both prior to, and following, EndoH treatment (Figure 3A, lanes 3 and 4). Specifically, in untreated lysates a lower form of gp41 (~41 kDa) was observed; furthermore, in EndoH-treated lysates the majority of gp41 collapsed to about 39 kDa, suggesting EndoH sensitivity. These observations support the ER retention of Env upon TBC1D20 overexpression. The low but detectable level of EndoH-sensitive gp41 present in TBCID20-expressing cells may indicate the premature cleavage of gp160. This may be the result of abnormal retention of furins in the ER, resulting in untimely cleavage of gp160. Alternatively, the ER localization of cleaved Env may result from its retrograde transport back into the ER. Another explanation is that this form of EndoH-sensitive gp41 may represent gp41 molecules that had normally proceeded through the Golgi yet retained high-mannose oligosaccharide side chains, since it has been shown that gp120 and gp41 contain both high-mannose (EndoH-sensitive) and terminally processed (EndoH-resistant) carbohydrate chains [34]. In this case, the presence of high-mannose side chains does not necessarily indicate a pre-Golgi localization. However, even in this scenario, the fact that TBC1D20 overexpression resulted in almost a full elimination of EndoH-resistant gp41 forms (Figure 3A, compare lanes 1 and 3) suggests TBC1D20-mediated impairment of Env trafficking. Of note, the anti-gp41 Chassie antibody also detected ~55 kDa bands in PNGase F-treated extracts (Figure 3B). In principle, these bands can correspond to deglycosylated from of gp120 since N-linked glycans comprise about 50% of the mass of gp120 [35] and as PNGase F treatment shifts gp120 MW to ~60 kDa [36]. Indeed, these bands also reacted with anti-gp120 antibody when the membrane was re-probed (data not shown). However, since the Chassie antibody does not react with gp120 (our observations and see [37]), we cannot exclude the possibility that the antibodies cross-reacted with a deglycosylated protein that is not related to HIV Env.

**Figure 4:**
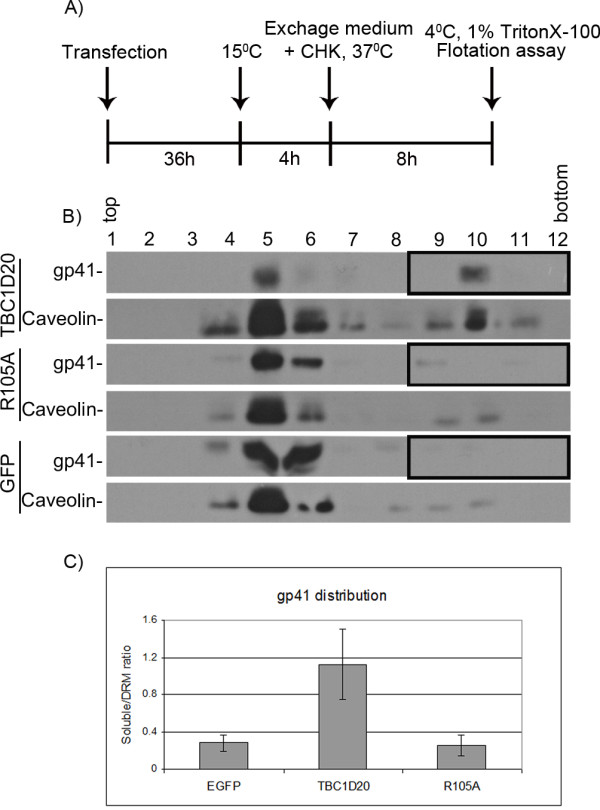
**TBC1D20 overexpression interferes with the association of HIV Env with DRMs**. (A) Timeline of experimental procedure (CHK, cyclohexamide). (B) Equilibrium flotation centrifugation assay to determine gp41 distribution in soluble and detergent-resistant cellular fractions. Cells (~107 cells/10 cm) overexpressing the indicated GAPs (fused to GFP) or GFP were fractionated and analyzed by Western blotting, using anti- gp41 and caveolin antibodies. Top and Bottom designate the top and bottom gradient fractions, respectively. The ratio between gp41 levels in the soluble fractions (fractions 9-12) and in the detergent-resistant fractions (fractions 4-7) was determined by densitometry (C), and is represented as the mean ± SEM (*n *= 3).

To achieve efficient assembly, Env proteins need to localize to microdomains at the plasma membrane, named detergent-resistant membranes (DRMs) [16,17]. The here-described TBC1D20-induced alterations of Env trafficking are expected to reduce its localization at these sites. To investigate this, we monitored the distribution of gp41 between DRMs and soluble fractions in cells overexpressing TBCID20, R105A (both fused to GFP), or GFP alone, using equilibrium flotation centrifugation [38]. Caveolin, expressed in 293T cells and localized to DRMs, served as a marker for DRMs-enriched fractions [16,17]. 293T cells were transfected (as described in Figure 2A, with four fold DNA amounts), 36 h post transfection the cultures were moved from 37°C to 15°C for an additional 4 h to retain proteins in the ER [39]. Then, cyclohexamide (50 μg/ml) was added to inhibit protein synthesis, and the cultures were reincubated at 37°C (for 8 h) to synchronize the ER exit of proteins. Subsequently, cells were extracted at 4°C with Triton X-100, and the lysates were separated on sucrose gradients (Figure 4A). Fractions of the gradients were analyzed by Western blotting, using anti-gp41 and anti-caveolin antibodies (Figure 4B). This analysis revealed that overexpression of TBC1D20, but not controls, resulted in marked reduction in DRM-localized gp41 (Figure 4B; fractions 4-7), while the remaining gp41 was localized to soluble fractions (Figure 4B; fractions 9-12, boxed). The average ratio between gp41 levels in soluble versus detergent-resistant fractions, calculated by densitometry was approximately five times higher for TBC1D20-overexpression, compared to the controls (*n *= 3, Figure 4C). These results are in line with Env localization to the earlier membrane compartments of the secretory pathway upon TBC1D20 overexpression (see Figure 3); further suggesting that TBC1D20 overexpression reduces the trafficking of Env towards the assembly sites at the plasma membrane. The residual Env levels found in virions and DRMs upon TBC1D20 overexpression (Figure 2A and 4) could result from early Env targeting to the plasma membrane, before TBC1D20 blockage and/or the outcome of residual Env trafficking through an unconventional secretion pathway, induced by ER stress [12,13].

Overall, TBC1D20 may affect virus replication cycle either directly, as in the case of the hepatitis C virus where interaction of TBC1D20 with the viral protein NS5A is required for viral RNA replication [9,40]; or indirectly, as described here for HIV-1 and recently for herpes simplex virus [41], where TBC1D20 affects the ER-trafficking of the viral envelope glycoproteins.

To summarize, here, we showed that enhancement of TBC1D20 activity, a negative regulator of Rab1 function, perturbs HIV-1 infectivity. This adds the TBC1D20/Rab1 axis to other identified factors of the secretory pathway that influence HIV replication cycle, such as Rab5, 6a, 7, 9 and 11a, [1,42-45]; and places TBC1D20 in the network of host regulators of the late stages of HIV infection.

## Competing interests

The authors declare that they have no competing interests.

## Authors' contributions

DN participated in the design of the study, performed the experiments and wrote the manuscript. EHS, ME and EB conceived of the study, participated in the design of the study, and helped to draft and edit the manuscript. All authors read and approved the final manuscript.
